# Maternal DNA Methylation Regulates Early Trophoblast Development

**DOI:** 10.1016/j.devcel.2015.12.027

**Published:** 2016-01-25

**Authors:** Miguel R. Branco, Michelle King, Vicente Perez-Garcia, Aaron B. Bogutz, Matthew Caley, Elena Fineberg, Louis Lefebvre, Simon J. Cook, Wendy Dean, Myriam Hemberger, Wolf Reik

**Affiliations:** 1 Blizard Institute, Barts and The London School of Medicine and Dentistry, QMUL, London E1 2AT, UK; 2 Epigenetics Programme, Babraham Institute, Cambridge CB22 3AT, UK; 3 Department of Medical Genetics, Life Sciences Institute, University of British Columbia, Vancouver, BC V6T 1Z3, Canada; 4 Signalling Programme, Babraham Institute, Cambridge CB22 3AT, UK; 5 Centre for Trophoblast Research, University of Cambridge, Cambridge CB2 3EG, UK; 6 The Wellcome Trust Sanger Institute, Cambridge CB10 1SA, UK

## Abstract

Critical roles for DNA methylation in embryonic development are well established, but less is known about its roles during trophoblast development, the extraembryonic lineage that gives rise to the placenta. We dissected the role of DNA methylation in trophoblast development by performing mRNA and DNA methylation profiling of *Dnmt3a/3b* mutants. We find that oocyte-derived methylation plays a major role in regulating trophoblast development but that imprinting of the key placental regulator *Ascl2* is only partially responsible for these effects. We have identified several methylation-regulated genes associated with trophoblast differentiation that are involved in cell adhesion and migration, potentially affecting trophoblast invasion. Specifically, trophoblast-specific DNA methylation is linked to the silencing of *Scml2*, a Polycomb Repressive Complex 1 protein that drives loss of cell adhesion in methylation-deficient trophoblast. Our results reveal that maternal DNA methylation controls multiple differentiation-related and physiological processes in trophoblast via both imprinting-dependent and -independent mechanisms.

## Highlights

• Oocyte-derived DNA methylation is an important regulator of trophoblast transcription
• DNA methylation controls trophoblast cell adhesion
• Silencing of Polycomb gene *Scml2* is necessary for normal trophoblast development

## Graphical Abstract

**Figure:**
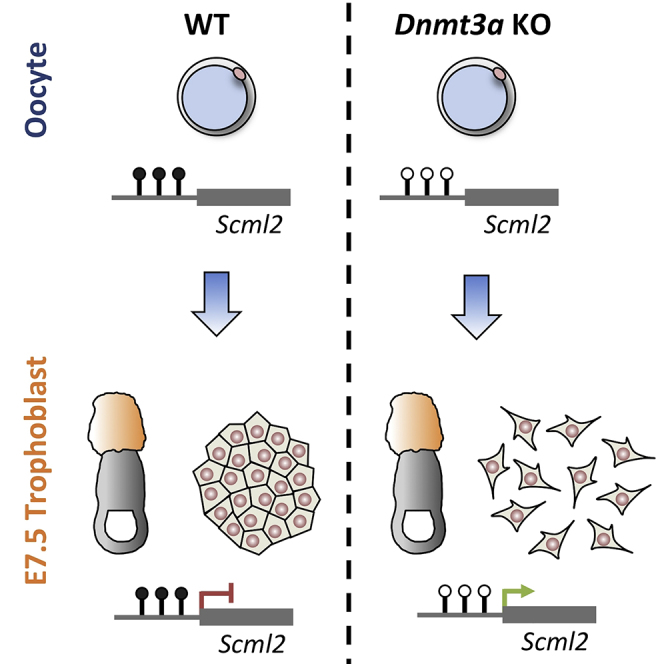

## Teaser

Branco et al. dissect the role of DNA methylation in mouse trophoblast development through genome-wide profiling of methylation-deficient mutants. DNA methylation marks carried over from the oocyte play a major role in trophoblast development and cell adhesion, which is partly dependent on silencing of the Polycomb gene *Scml2*.

## Introduction

Fertilization marks the start of a cascade of rapid epigenetic changes which, coupled to an intricate network of signaling and transcriptional events, ultimately lead from a totipotent zygote to a myriad of differentiated tissues that comprise the embryo as well as supporting extraembryonic tissues. DNA methylation plays essential roles during this time, mainly by mediating silencing of specific genes and transposable elements. Importantly, while genome-wide DNA methylation erasure occurs after fertilization, key genomic regions are kept methylated, including imprinting control regions (ICRs) and murine intracisternal A-particle (IAP) retrotransposons (Lane et al., 2003, Smith et al., 2012). This epigenetic reprogramming phase is followed by de novo DNA methylation post-implantation, which helps to establish and cement tissue-specific expression programs, thereby driving cell differentiation and organogenesis.

In mammals, three DNA methyltransferases (DNMTs) are responsible for establishing and maintaining DNA methylation profiles: DNMT1 is mainly involved in the maintenance of methylation patterns during replication, whereas DNMT3A and DNMT3B have de novo methylation activity. Mouse knockout (KO) models have shown that all three enzymes are essential for correct embryonic development: *Dnmt1* and *Dnmt3b* KOs are embryonic lethal (Li et al., 1992, Okano et al., 1999), whereas *Dnmt3a* KO mice die postnatally (Okano et al., 1999). The combined double KO (DKO) of *Dnmt3a* and *Dnmt3b* has a more severe phenotype than either single KO, with embryos dying at around embryonic day 10.5 (E10.5) (Okano et al., 1999). Importantly, conditional deletion of *Dnmt3a* in the oocyte is sufficient to halt embryonic development at E10.5 (Kaneda et al., 2004), showing that maternal methylation is critical for developmental progression. Maternal KO of *Dnmt3l*, a catalytically inactive co-factor that interacts with DNMT3A for the establishment of DNA methylation in the oocyte, displays a very similar phenotype (Bourc'his et al., 2001).

While the role of DNA methylation in the development of the embryo is well established (Auclair et al., 2014), its importance in the development of the extraembryonic trophoblast lineage remains less clear. Notably, trophoblast tissues are largely hypomethylated when compared with embryonic tissues, in particular at repeat elements, and embryos derived by nuclear transfer from ESCs lacking all three active DNMTs can contribute to extraembryonic tissues when aggregated with wild-type (WT) embryos (Sakaue et al., 2010). On the other hand, conceptuses from *Dnmt3l*-null mothers display morphogenic defects across all layers of the placenta (Arima et al., 2006). To date, comprehensive molecular characterization of the DNA methylation and gene expression alterations linked to these defects is lacking. While they have been largely attributed to the loss of imprinted gene expression, some methylation marks outside of imprinting are also carried over from the oocyte to the blastocyst stage (Smallwood et al., 2011), but have not been functionally explored. Indeed, of 1,329 CpG islands (CGIs) that are hypermethylated in oocytes relative to sperm, only 23 are associated with known ICRs (Kobayashi et al., 2012). In this study, we have performed mRNA sequencing (mRNA-seq) and whole-genome bisulfite sequencing (BS-seq) on trophoblast from *Dnmt3a/3b* KO mice. We show that the prevailing phenotype is explained by the absence of maternal methylation marks. However, failure to establish correct imprinted gene expression does not explain all observed transcriptional changes. Our data suggest that maternal DNA methylation plays critical roles in the control of cell adhesion in trophoblast giant cells (TGCs) and in the formation of syncytiotrophoblast (SynT).

## Results

### Absence of Oocyte DNA Methylation Leads to Cell Adhesion Defects

**Figure 1:**
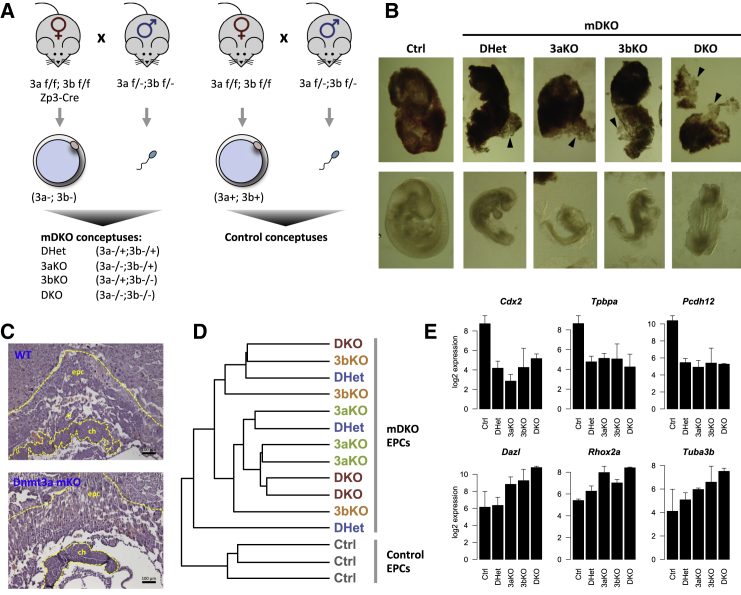
Oocyte Methylation Is a Major Regulator of Trophoblast Gene Expression (A) Females carrying floxed (f) alleles for *Dnmt3a* and *Dnmt3b* as well as a *Zp3*-driven Cre transgene were crossed to heterozygous males, yielding four different genotypes collectively referred to as mDKO, due to the absence of methylation in the oocyte; conceptuses from females without the *Zp3*-Cre transgene were used as controls. (B) Maternal deletion of *Dnmt3a/3b* results in trophoblast defects at E9.5 (top) characterized by loss of adhesion of TGCs (arrowheads), with no apparent difference in phenotype between different post-zygotic genotypes. In contrast, DKO embryos are more severely affected than DHet embryos (bottom). Images are not on the same scale. (C) H&E staining of paraffin-embedded sections shows that *Dnmt3a* mKO trophoblast lacks the labyrinthine layer that is otherwise seen developing in WT trophoblast (marked by an asterisk); the TGC layer is less dense in *Dnmt3a* KO trophoblast, possibly due to cell adhesion defects. ch, chorion; epc, ectoplacental cone. (D) Hierarchical clustering of mRNA-seq data from E7.5 EPCs reveals segregation of mDKO and Ctrl genotypes but no further differentiation of individual mDKO genotypes. (E) mRNA-seq expression values for examples of deregulated genes common to all mDKO genotypes (top), and genes controlled by post-zygotic DNA methylation (bottom). Error bars represent SD. See also Figure S1.

To study the role of DNA methylation in trophoblast development, we used female mice carrying conditional alleles for both *Dnmt3a* and *Dnmt3b* (Dodge et al., 2005, Kaneda et al., 2004), as well as a *Zp3-Cre* transgene; these were crossed to double heterozygous males, i.e., *Dnmt3a*^*+/−*^;*Dnmt3b*^*+/−*^ (Figure 1A). Deletion of *Dnmt3a* and *Dnmt3b* driven by *Zp3* expression yields oocytes that lack both enzymes (and virtually all DNA methylation) (Kaneda et al., 2010, Shirane et al., 2013). We will therefore refer to the group of genotypes resulting from this cross collectively as maternal DKOs (mDKOs), and the individual genotypes derived from this cross as such: DHet for *Dnmt3a*^*−/+*^;*Dnmt3b*^*−/+*^, 3aKO for *Dnmt3a*^*−/−*^;*Dnmt3b*^*−/+*^, 3bKO for *Dnmt3a*^*−/+*^;*Dnmt3b*^*−/−*^, and DKO for *Dnmt3a*^*−/−*^;*Dnmt3b*^*−/−*^. To generate a control cohort, we made a separate cross using females without the *Zp3-Cre* transgene (Figure 1A). For simplicity, we will refer to these genotypes as control (Ctrl) genotypes and will not distinguish between the various combinations of WT homozygous and heterozygous alleles generated by this cross.

We first dissected conceptuses at E9.5 for morphological characterization. As previously described (Okano et al., 1999), DKO embryos were severely developmentally delayed, with few defined somites and open neural tube, among other defects, whereas other genotypes exhibited less pronounced abnormalities (Figure 1B). However, to our surprise trophoblast tissues showed a very consistent phenotype across all genotypes of the mDKO cohort, with no obvious additional defects being observed in DKO trophoblast over DHet trophoblast (Figure 1B). The most prominent characteristic of these tissues was a reduction in the adhesion of TGCs that make up the outermost lining of the implantation site, as these cells were easily dissociated from the remaining tissue when compared with control trophoblast. Maternal deletion of *Dnmt3a* was sufficient to produce the same phenotype. Histological analysis of *Dnmt3a* maternal knockout (mKO) trophoblast at E9.5 revealed defects similar to those described for *Dnmt3l* mKO trophoblast (Arima et al., 2006, Bourc'his et al., 2001). Namely, *Dnmt3a* mKOs had a defect in chorio-allantoic fusion that in turn led to a failure in development of the labyrinthine layer, which can be made out in WT trophoblast by finger-like invaginations of the allantoic mesoderm into the chorionic ectoderm layer forming well-defined fetal blood spaces (Figure 1C). In addition, while the TGC layer appeared enlarged, this was mostly a result of reduced tissue density, as there was a notable increase in extracellular space in this layer (Figures 1C and S1A). It is possible that such spacing only becomes apparent as a result of the histological preparation, but given that WT and mKO tissues were processed in parallel and embedded in the same paraffin block, this is likely to be an expression of the loss of cell adhesion seen in dissected tissues. Cellular adhesion plays key roles in placental development, regulating trophoblast invasiveness into the maternal decidua (Harris et al., 2009, Sutherland et al., 1993). Importantly, dysregulation of adhesion molecules is commonly observed in placental disorders, including pre-eclampsia and intrauterine growth restriction (Harris et al., 2009, Pollheimer and Knöfler, 2005). It is therefore plausible that the developmental arrest of methylation-deficient conceptuses is due, at least in part, to alterations in the cellular adhesion profile of trophoblast cells.

### mRNA Profiling Reveals Genes Controlled by Oocyte Methylation

To gain deeper insights into these hypomethylation-induced changes, we profiled the transcriptome of mDKO and Ctrl trophoblasts. To compare structurally similar tissues and identify primary, causative aberrations in the gene expression patterns, we used E7.5 ectoplacental cones (EPCs), at which point mDKO conceptuses were visually indistinguishable from Ctrl conceptuses. We performed mRNA-seq of three EPCs from each genotype group. Interestingly, hierarchical clustering of the data mirrored our phenotypic observations: Ctrl EPCs clustered together, away from a large group of mDKO EPCs, with no individual genotype therein being discernible from the others (Figure 1D). Accordingly, we identified 368 differentially expressed (DE) genes between Ctrl and DHet EPCs, whereas comparison of DHet with 3aKO or 3bKO genotypes yielded only 4 and 6 DE genes, respectively. This strongly suggests that nearly all transcriptional effects are due to oocyte methylation deficiency. Interestingly, we did identify 45 DE genes between DKO and DHet EPCs. As previously described for embryonic tissues (Auclair et al., 2014, Borgel et al., 2010, Hackett et al., 2012), these include germline-specific genes such as *Dazl*, *Rhox2a*, and *Tuba3b* (Figure 1E). These genes are repressed by de novo methylation, as post-zygotic deletion of *Dnmt3a/3b* is sufficient to cause their upregulation (Figure S1B).

To focus on the group of oocyte methylation-dependent genes, we selected 137 genes that passed stringent criteria for differential expression in all mDKO genotypes when compared with Ctrl EPCs (Table S1). These “mDKO DE genes” were made up of 39 upregulated and 98 downregulated genes, and showed very similar expression levels in all mDKO genotypes (Figure S1C), suggesting that they are solely dependent on oocyte methylation and not on the presence of DNMTs post-fertilization. Concordantly, post-zygotic deletion of *Dnmt3a/3b* has no effect on the expression of mDKO DE genes (Figure S1B). Among the mDKO DE genes were transcription factors and markers relevant to trophoblast development, such as *Cdx2*, *Tpbpa*, and *Pcdh12* (Figure 1E, RT-qPCR validation in Figure S1B). Given that *Cdx2* is a key trophoblast stem cell (TSC) transcription factor, we asked whether the TSC niche was affected in *Dnmt3a* mKO mutants by performing immunofluorescence for CDX2 on sections of E7.5 conceptuses. We found that CDX2 depletion only occurred in the EPC and that the extraembryonic (chorionic) ectoderm, which harbors the TSC niche, remained unaffected (Figure S1D), suggesting that loss of CDX2 is unrelated to deregulation of the stem cell compartment, but critically affects the diploid core of the EPC. Key markers of placental development were also affected, including the spongiotrophoblast marker *Tpbpa* and the glycogen cell precursor marker *Pcdh12* (Figures 1E and S1B). Notably, TGC markers such as *Prl3d1*/*Pl1* were unaffected (Figure S1B), suggesting that the observed phenotypic alterations are not due to major defects in the differentiation of this placental cell type.

### Loss of *Ascl2* Imprinting Only Partially Explains Alterations in mDKO Trophoblast

**Figure 2:**
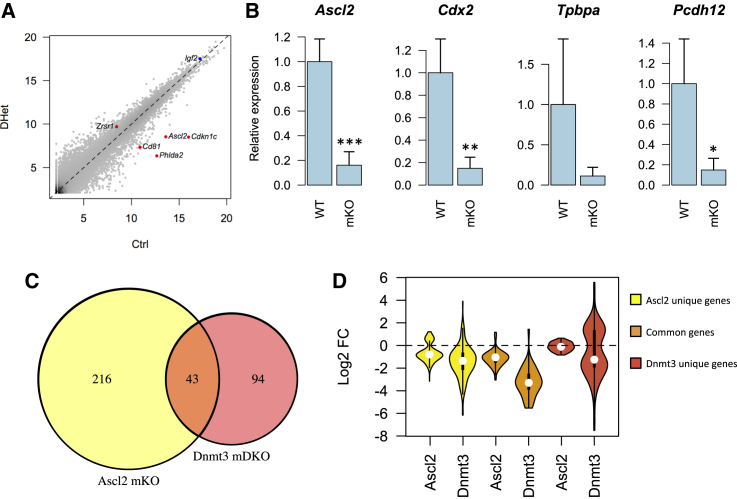
*Ascl2* Depletion Does Not Drive the Bulk of Gene Expression Alterations in mDKO EPCs (A) mRNA-seq data reveal deregulated maternally controlled imprinted genes in DHet EPCs (red), whereas a paternally controlled imprinted gene is unchanged (*Igf2*, blue). (B) RT-qPCR data from *Ascl2* WT and mKO E7.5 EPCs shows that some key genes deregulated in mDKO EPCs are driven by *Ascl2* downregulation (∗p < 0.05, ∗∗p < 0.01, ∗∗∗p < 0.001; t test). Error bars represent SD. (C) Differential expression analysis from mRNA-seq of *Ascl2* mKO EPCs reveals that only a minority of *Dnmt3* mDKO DE genes are explained by *Ascl2* repression. (D) Log2 fold change in expression between WT and *Ascl2* or *Dnmt3* mutant EPCs for each grouping of genes defined in (C). *Ascl2*-dependent genes display similar expression changes in *Dnmt3* mDKO EPCs, whereas *Dnmt3* unique DE genes are unchanged in *Ascl2* mKO EPCs. See also Figure S2.

Oocyte methylation controls several ICRs that are essential for maternal regulation of imprinted genes, which are important for both embryo and trophoblast development. As previously suggested (Arima et al., 2006), maternal effects on trophoblast development may therefore be a result of loss of specific imprinted genes. To test this hypothesis, we first identified known methylation-dependent imprinted genes within our mDKO DE gene list. Of 79 imprinted genes (paternal and maternal) in our mRNA-seq data, 59 were robustly expressed in EPCs from at least one of the genotypes. However, only five were consistently altered in all mDKO genotypes: *Zrsr1*, *Cd81*, *Ascl2*, *Phlda2*, and *Cdkn1c* (Figure 2A). *Zrsr1* (also known as *U2af1-rs1*) was upregulated in mDKO EPCs but is unlikely to be involved in the phenotype of mDKO conceptuses, as mice with paternal disomy of chromosome 11 (where *Zrsr1* lies) are viable (Cattanach and Kirk, 1985). *Cd81*, *Cdkn1c*, *Phlda2*, and *Ascl2*, which are all part of the same imprinting cluster, were all robustly downregulated in mDKO EPCs, as lack of oocyte methylation leads to activation of the non-coding transcript *Kcnq1ot1* on the maternal allele, which is known to drive silencing of genes in its vicinity (Peters and Robson, 2008). *Cd81* KO mice are viable (Maecker and Levy, 1997), and both *Cdkn1c* and *Phlda2* KO placentas are enlarged and show an expansion of the spongiotrophoblast layer, which is a very different phenotype from that observed in mDKOs (Frank et al., 2002, Takahashi et al., 2000). However, trophoblast from maternal KO of *Ascl2* (also known as *Mash2*) has a severe phenotype mainly characterized by a lack of spongiotrophoblast formation, which leads to embryonic lethality at around E10 (Guillemot et al., 1994). Given the similarity in phenotype timing to the mDKO trophoblast, as well as the downregulation of spongiotrophoblast marker *Tpbpa* observed in both models, we decided to test whether *Ascl2* downregulation was driving the transcriptional changes seen in mDKO EPCs. For this purpose, we used an *Ascl2-lacZ* knockin mouse line (Tanaka et al., 1999) to generate mKO conceptuses of *Ascl2*. We first performed histological analysis at E9.5, which revealed that *Ascl2* mKOs had a reduced or absent labyrinthine layer despite having completed chorio-allantoic fusion, and had an enlarged TGC layer (Figure S2A), as previously described (Guillemot et al., 1994). However, unlike *Dnmt3a* mKO trophoblast, the TGC layer expansion did not involve a significant increase in extracellular space (Figure S2B), inferring that TGC cell adhesion is largely intact in *Ascl2* mKO mutants. We then isolated E7.5 EPCs from *Ascl2* WT and mKO conceptuses for RT-qPCR analyses. We confirmed that *Ascl2* repression leads to *Tpbpa* downregulation, but also found drastic downregulation of *Cdx2* and *Pcdh12*, similar to that seen in *Dnmt3* mDKO EPCs (Figure 2B). We then extended this analysis by performing mRNA-seq on control and *Ascl2* mKO EPCs. We found that, while 43 genes were commonly deregulated between *Ascl2* mKO and *Dnmt3* mDKO EPCs, there were 94 DE genes that were unique to the *Dnmt3* mDKOs (Figure 2C). Surprisingly, we also found 216 genes seemingly only deregulated in *Ascl2* mKOs. However, when we analyzed the expression of these genes in *Dnmt3* mDKO EPCs, we found that they displayed expression changes very similar to those seen in *Ascl2* mKOs (Figure 2D), but had not passed our stringent criteria for differential expression calling. Importantly, genes deregulated only in the *Dnmt3* mDKO EPCs did not display substantial changes in expression in *Ascl2* mKOs (Figure 2D), demonstrating that these are indeed *Ascl2*-independent effects. Our data suggest that the majority of transcriptional alterations in mDKO trophoblast are independent of imprinting of a key regulator of placental development. While we cannot completely rule out that the combined loss of imprinting at other loci may drive the gene expression changes seen in mDKOs, it is likely that maternally derived methylation marks outside of ICRs play a major role in trophoblast gene regulation.

### mDKO-Affected Genes Are Involved in Trophoblast Development and Adhesion

**Figure 3:**
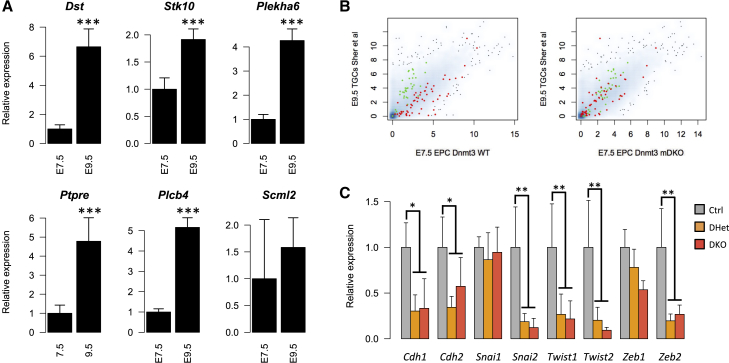
mDKO DE Genes Are Associated with Trophoblast Differentiation (A) Several *Dnmt3*-specific genes affected in mDKO EPCs become upregulated during trophoblast development, as revealed by RT-qPCR of E7.5 and E9.5 WT trophoblast. (B) Expression of mDKO DE genes in E9.5 TGCs relative to E7.5 EPCs. mRNA-seq data from E9.5 TGCs (Sher et al., 2013) were plotted against our mRNA-seq data for Ctrl (left) or mDKO (right) E7.5 EPCs. Genes upregulated in mDKO EPCs (green) have increased expression in TGCs relative to WT E7.5 EPCs. Similarly, mDKO downregulated genes (red) tend to have lower expression in TGCs. When plotted against mDKO EPC data, the expression of mDKO DE genes is much more comparable with that seen in TGCs. (C) RT-qPCR of EMT-associated genes in E9.5 trophoblast reveals that mDKO trophoblasts do not exhibit a classic EMT phenotype, although *Cdh1* is downregulated. Error bars represent SD. ∗p < 0.05, ∗∗p < 0.01, ∗∗∗p < 0.001; t test comparing E9.5 with E7.5 (A), or ANOVA with post hoc tests comparing control (Ctrl) and mDKO genotypes (C). See also Figure S3.

We decided to focus on the group of 94 genes that were affected in *Dnmt3* mDKO but not *Ascl2* mKO EPCs. Gene ontology (GO) analysis revealed that this *Dnmt3*-specific gene list was enriched for genes involved in signal transduction (e.g., *Ephb2*, *Stk10*, *Pik3ap1*, *Ptpn3*) and the regulation of guanosine triphosphatases (GTPases) (e.g., *Asap1*, *Rgs3*, *Arhgef4*, *Rasa4*) (Table S2), whereas no such enrichment was seen in *Ascl2*-specific genes (Table S3). GTPases control many key cellular processes, including focal adhesion and migration/invasion (Menke and Giehl, 2012), which is consistent with the cellular adhesion defect observed in mDKO trophoblast. Notably, numerous other genes involved in cell adhesion were found to be deregulated in mDKO, such as *Itga7*, *Flnc*, *Dbnl*, and *Plxna1*. Although the GO term “cell adhesion” did not reach significance in our analysis, we noted that many deregulated genes with known roles in cell adhesion and migration lack this annotation (e.g., *Asap1*, *Rasa4*, *Srgap3*, *Spry1*). Acquisition of an invasive phenotype is a key component of the differentiation process of TGCs (Hemberger et al., 2003, Hunkapiller et al., 2011). In line with this, *Ephb2*, a receptor tyrosine kinase that activates Rho family GTPases and is involved in the formation of secondary TGCs (El-Hashash and Kimber, 2006), was downregulated in mDKO EPCs. Other deregulated genes involved in trophoblast differentiation included *Gata3* (Ralston et al., 2010), *Gjb5* (Kibschull et al., 2014), *Dlx3* (Morasso et al., 1999), and *Alkbh1/2700073G19Rik* (Pan et al., 2008). This prompted us to ask whether mDKO-affected genes were generally associated with trophoblast differentiation. Genes annotated with GO terms associated with trophoblast or placental development were not significantly enriched (although we again found this annotation to be incomplete). However, five out of six tested genes showing upregulation in mDKO EPCs were found to increase in expression during differentiation in vivo (Figure 3A), and similar results were obtained during in vitro differentiation of TSCs (Figure S3). To expand on these observations using an annotation-independent approach, we examined the behavior of mDKO-affected genes in expression data from E9.5 WT TGCs (Sher et al., 2013) and found that most of these genes are indeed differentially expressed relative to WT E7.5 EPCs (Figure 3B). Moreover, their expression in mDKO E7.5 EPCs was well correlated with the expression profile of E9.5 TGCs (Figure 3B), indicative of precocious TGC differentiation in mDKO trophoblast. Although some TGC markers (such as *Prl3d1*) are unchanged in mDKO EPCs (Figure S1B), it is likely that deregulation of these TGC-associated genes affects the function of this cell population.

Trophoblast invasion and placental development critically depend on epithelial-to-mesenchymal transition (EMT) (Kokkinos et al., 2010, Parast et al., 2001, Sutherland, 2003). Notably, apart from signal transduction and GTPase regulators, other mDKO-deregulated genes with known roles in EMT included *Mmp15* (Tao et al., 2011) and *Grhl2* (Cieply et al., 2012). However, genes widely involved in EMT (e.g., *Cdh1*, *Snai1*, *Zeb1*) were not significantly altered in our E7.5 mRNA-seq data. To test whether an overt EMT phenotype expressed itself at a later developmental stage, we measured the expression of key EMT-associated genes in E9.5 Ctrl and mDKO trophoblast (Figure 3C). While we did observe a prominent decrease in the classic epithelial marker *Cdh1* (E-cadherin), this was unexpectedly accompanied by a comparable reduction in the mesenchymal marker *Cdh2* (N-cadherin). Similarly, while EMT is commonly driven by the expression of *Snai1/2* (*Snail* and *Slug*), *Twist1/2*, and *Zeb1/2*, we found either no change or a robust decrease in the expression of these genes (Figure 3C). These results indicate that the cell adhesion changes observed in mDKO trophoblast do not display the characteristics of a classic EMT and may be driven by independent or non-canonical pathways.

Contrary to what has been previously suggested (Sakaue et al., 2010), these data show that oocyte methylation, directly or indirectly, regulates genes that are important for trophoblast differentiation and function. Notably, genes involved in signal transduction pathways that control adhesion and migration are particularly affected.

### Methylation-Deficient TSCs Display Cell Adhesion Defects

**Figure 4:**
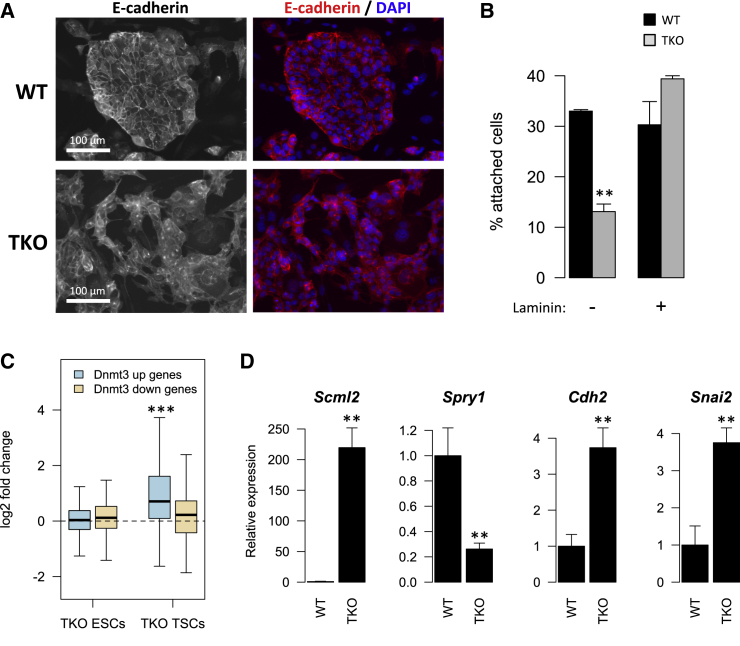
Loss of DNA Methylation Drives a Cell Adhesion Defect in TSCs (A) E-cadherin staining of WT and TKO TSCs highlights that TKO TSCs do not display the typical epithelial colony morphology of ESCs, appearing to exhibit reduced cell adhesion and increased migration. (B) TKO TSCs also have reduced adhesion capacity to cell culture wells, but only in the absence of the extracellular matrix component laminin. (C) Gene expression differences (log2 fold change) between TKO and WT ESCs or TSCs for all mDKO-deregulated genes. *Dnmt3* upregulated genes are derepressed in TKO TSCs (but not ESCs), consistent with a direct role of DNA methylation in their regulation, whereas downregulated genes are unchanged. (D) RT-qPCR on TKO TSCs shows expression changes consistent with those seen in mDKO trophoblast (*Scml2* and *Spry1*) and raised expression of mesenchymal markers (*Cdh2* and *Snai2*). Bar plot error bars represent SD. ∗∗p < 0.01, ∗∗∗p < 0.001; t test comparing WT with TKO TSCs (B and D), or one-sample t test (C). See also Figure S4.

To further investigate the link between DNA methylation and cellular adhesion, we cultured TSCs null for all three active DNA methyltransferases (*Dnmt1*, *Dnmt3a*, and *Dnmt3b*) and compared them with WT TSCs; both being derived from nuclear transfer embryos (Sakaue et al., 2010). We observed that WT cells grew as flat epithelial colonies with sharp boundaries as is characteristic of TSCs, whereas triple-knockout (TKO) TSCs did not form distinct colonies but instead exhibited a much more disorganized morphology and a notable loss of discrete colony margins (Figure 4A). In WT TSCs, E-cadherin delineated cell-cell junctions within colonies, whereas in TKO TSCs such junctions were less frequent and instead fibroblast-like cytoplasmic protrusions were visible, suggestive of an increased migratory capacity. Furthermore, using an assay to measure cell adhesion to tissue culture wells, we found that TKO TSCs were less adherent than WT cells on uncoated wells (Figure 4B). Interestingly, the difference could be rescued by laminin, suggesting that the adhesion defect can be overcome by changing the ECM/substrate composition. These differences were not due to cell differentiation, as TKO TSCs retained expression of key TSC markers (Figures S4 and 6G). In addition, TSCs derived from *Ascl2* mKO trophoblast do not display any morphological abnormalities compared with their WT counterparts (A.B.B. and L.L., unpublished data), ruling out a role for the loss of *Ascl2* imprinting in the TKO TSC phenotype.

To test whether loss of cell adhesion in TKO TSCs was driven by deregulation of the same genes identified in mDKO trophoblast, we plotted the expression changes of mDKO DE genes in TKO TSCs using published microarray data (Figure 4C) (Sakaue et al., 2010). We found that mDKO upregulated genes are also derepressed in TKO TSCs, supporting a role for DNA methylation in their silencing. In contrast, mDKO downregulated genes were unaffected, suggesting that they are likely indirect effects that occur in the context of trophoblast differentiation. Interestingly, in contrast to TKO TSCs, TKO ESCs showed no changes in the expression of mDKO upregulated genes (Figure 4C), indicating that TSC-specific transcription factors are required to activate these genes. In line with this hypothesis, we found that mDKO upregulated genes (but not downregulated genes) were enriched for sites bound by both ELF5 and TFAP2C (p = 1.3 × 10^−10^) (Latos et al., 2015).

Using RT-qPCR on WT and TKO TSCs we confirmed the derepression of *Scml2*, which was the top mDKO upregulated gene (Figure 4D). Equally concordant with the in vivo data was the downregulation of *Spry1*, which is a major EGFR signaling regulator that controls cell adhesion and migration (Mekkawy et al., 2014). We also detected an increase in the expression of the mesenchymal markers *Cdh2* and *Snai2* (Figure 4D), although this was not accompanied by significant changes in *Cdh1* or *Snai1* (Figure S4), suggesting that, similarly to the mDKO trophoblast, canonical EMT is not involved in the loss of cell adhesion in TKO TSCs.

These results further support a critical role for DNA methylation in the regulation of cell adhesion in trophoblast.

### A Subset of mDKO Upregulated Genes Are Controlled by DNA Methylation

**Figure 5:**
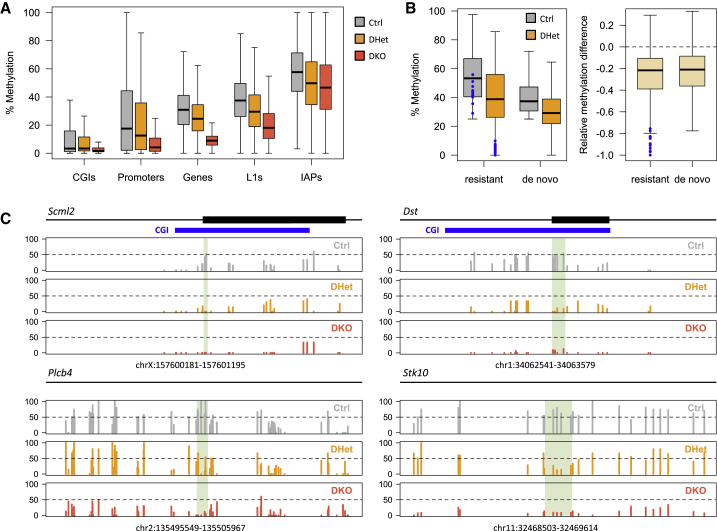
DNA Methylation Differences Are Associated with DE Genes (A) DNA methylation profiling by BS-seq shows that DHet EPCs have a slight genome-wide reduction of DNA methylation. (B) CGIs methylated (>25%) in Ctrl EPCs were separated into reprogramming-resistant (>25% methylation in blastocyst; Kobayashi et al., 2012) or de novo methylated (<15% methylation in blastocyst). Methylation levels in Ctrl and DHet EPCs (left) and the relative methylation change between the two (right) shows that both subsets are hypomethylated to a similar extent in DHet EPCs; maternally methylated ICRs (blue) undergo more extensive methylation loss. (C) BS-seq profiles of methylation levels at example loci containing DHet DMRs (highlighted in green) that are associated with genes displaying altered gene expression. See also Figure S5.

We next sought to identify which genes were likely to be regulated through the direct action of DNA methylation. To link gene expression changes with alterations in DNA methylation patterns, we performed whole-genome BS-seq of Ctrl, DHet, and DKO E7.5 EPCs. As expected, DKO EPCs showed deep and widespread hypomethylation across several genomic features, including gene promoters, gene bodies, and LINE1 retrotransposons (Figure 5A). IAP retrotransposons were largely resistant to demethylation, consistent with DNMT1 being sufficient to maintain DNA methylation at these sites (Arand et al., 2012).

DHet EPCs displayed a slight reduction in DNA methylation across most genomic features (Figure 5A). To test whether this was solely due to a loss of oocyte methylation, we compared our data with published data on CGI methylation in oocytes and blastocysts (Kobayashi et al., 2012). Most CGIs that are partially resistant to demethylation during pre-implantation development are methylated in oocytes (Figure S5) (Smallwood et al., 2011). We therefore asked whether these reprogramming-resistant CGIs were more likely to be hypomethylated in DHet EPCs when compared with de novo methylated CGIs. Our analysis shows that, overall, both groups of CGIs undergo a similar loss of methylation in DHet EPCs (Figures 5B and S5). In contrast, maternally methylated ICRs displayed a markedly more pronounced loss of methylation (Figure 5B). Therefore, it appears that the global hypomethylation seen in EPCs is a result of haploinsufficiency of *Dnmt3a* and/or *Dnmt3b* during the de novo methylation phase. However, the lack of correlation between the post-zygotic genotype and expression of mDKO DE genes (Figure S1C) argues against a major role of haploinsufficiency in driving the mDKO phenotype.

To exclude these small genome-wide differences in DNA methylation, we performed an unbiased search for differentially methylated regions (DMRs) between DHet and Ctrl EPCs that displayed at least a 20% methylation difference. We identified 6,685 DMRs, nearly all of which (96.6%) involved a loss of methylation in DHet EPCs and were seemingly not enriched at CGIs, promoters, gene bodies, or placental enhancers predicted from the mouse ENCODE project. Around 67% of DMRs had more than 80% methylation in WT oocytes (Kobayashi et al., 2012), whereas only 46% of randomly generated regions passed the threshold, suggesting an important contribution of oocyte methylation to these DMRs. We identified 59 DMRs lying within 20 kb of mDKO DE genes (excluding *Ascl2*-dependent effects), covering 35 out of 94 DE genes (see examples in Figure 5C). Surprisingly, these DMRs were associated with both upregulated (n = 14) and downregulated (n = 21) genes. To further probe which gene expression alterations were likely to be directly regulated by DNA methylation, we asked which genes were also affected in TKO TSCs. Notably, 6 out of 14 DMR-associated genes that were upregulated in mDKO EPCs were also more than 2-fold upregulated in TKO TSCs. Five of these genes (*Dst*, *Plekha6*, *Stk10*, *Ptpre*, and *Plcb4*) encoded an actin-binding protein, a phospholipid-binding adaptor, a protein kinase, a protein tyrosine phosphatase, and a phospholipase that are implicated in cell adhesion and signal transduction.

These data reveal that a number of oocyte-dependent DMRs are linked to genes deregulated in mDKO EPCs, a subset of which appear to be directly controlled by DNA methylation in a trophoblast-specific manner.

### *Scml2* Is Hypomethylated in mDKO EPCs and Affects Trophoblast Differentiation and Adhesion

**Figure 6:**
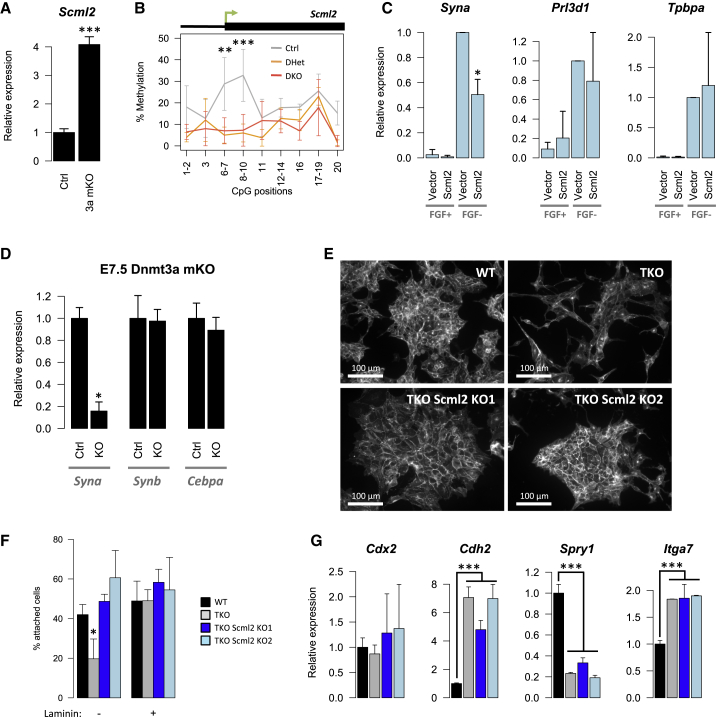
*Scml2* Is Controlled by DNA Methylation and Affects SynT Formation and Cell Adhesion (A) RT-qPCR of *Dnmt3a* mKO EPCs confirms that *Scml2* is controlled by oocyte methylation. (B) Methylation analysis by Sequenom MassARRAY in E7.5 male EPCs, confirming the DMR at an intragenic TSS of *Scml2*. Each data point may include more than one CpG from the amplicon, as indicated on the x axis. (C) RT-qPCR analysis of TSCs grown in FGF+ (TSC conditions) or FGF− (differentiation conditions) medium for 6 days, with or without *Scml2* overexpression. (D) Expression of *Syna* is reduced in *Dnmt3a* mKO EPCs, whereas markers of SynT-II *Synb* and *Cebpa* are unaffected. (E) E-cadherin staining of two independent *Scml2* knockout clones from TKO TSCs shows a rescue of the morphological alterations seen in TKO TSCs. (F) *Scml2* KO on TKO TSCs also rescues the defect in cell adhesion to cell culture wells in the absence of laminin. (G) RT-qPCR analysis of TKO *Scml2* KO clones shows maintained expression of the TSC marker *Cdx2*; the expression of genes involved in cell adhesion is not rescued upon *Scml2* deletion. Error bars represent SD. ∗p < 0.05, ∗∗p < 0.01, ∗∗∗p < 0.001; t test comparing WT and *Dnmt3a* mKO EPCs (A and D) or *Scml2*-expressing TSCs versus vector control (C), or ANOVA with post hoc tests comparing Ctrl with DHet/DKO (B) or TKO TSC lines with WT TSCs. See also Figure S6.

One of the DMRs we identified overlapped *Scml2* (Figure 5C), which was the most upregulated gene in mDKO EPCs, and was also highly upregulated in TKO TSCs (Figure 4D). SCML2 is a non-canonical member of the Polycomb Repressive Complex 1 (PRC1), which plays important epigenetic roles in the establishment of the male germline (Hasegawa et al., 2015, Luo et al., 2015). We therefore investigated whether *Scml2* silencing was important for trophoblast development and adhesion. Firstly, we confirmed that *Scml2* upregulation in mDKO EPCs was driven by the maternal deletion of *Dnmt3a* (Figure 6A). We then validated the DMR associated with *Scml2* using Sequenom MassARRAY, which showed a specific loss of methylation in DHet and DKO EPCs near an intragenic transcription start site (TSS) (Figure 6B). This alternative promoter overlaps a CGI (highlighted in Figure 5C) that is 95% methylated in WT oocytes (losing all methylation in *Dnmt3l*-null oocytes) and 34% methylated in blastocysts (Kobayashi et al., 2012), supporting the notion that *Scml2* methylation levels are carried over from the oocyte through the pre-implantation phase. Interestingly, maintenance of methylation and silencing of *Scml2* are specific to the trophoblast compartment, as methylation in the epiblast is lost and *Scml2* is expressed (Figure S6A). This pattern is also clearly observed when comparing ES and TS cells (Figure S6A). *Scml2* lies on the X chromosome, but while *Scml2* is unmethylated in X-containing sperm (Kobayashi et al., 2012), no placental *Scml2* expression is expected from the paternal allele in females due to imprinted X inactivation in mouse trophoblast. Accordingly, we found no difference in *Scml2* expression levels between male and female EPCs within Ctrl or mDKO genotypes. Furthermore, X inactivation appeared unaffected in female DHet EPCs, as *Xist* expression was unchanged. Importantly, the X-linked nature of *Scml2* makes its epigenetic regulation distinguishable from a genomic imprinting mechanism.

To test the effect of *Scml2* expression on trophoblast morphology and development, we overexpressed *Scml2* in TSCs (*Scml2* expression levels in Figure S6C). When cells were grown under stem cell conditions (fibroblast growth factor [FGF]+), we found no significant differences in morphology or cell adhesion (Figure S6B) upon overexpression of *Scml2*. There were also no detectable differences in the expression of key TSC markers (Figure S6C). However, after induction of TSC differentiation by removal of FGF from the medium, *Scml2* impaired the expression of Syncytin A (*Syna*), a marker of SynT-I (which interfaces the maternal blood), while no effect was seen on other differentiation markers, including markers of TGCs and SynT-II (Figure 6C and data not shown). This suggests that *Scml2* silencing is important for SynT-I formation in WT trophoblast. Accordingly, *Dnmt3a* mKO trophoblast also displayed reduced *Syna* expression at E7.5 but no alterations in SynT-II markers *Synb* and *Cebpa* (Figure 6D). While we found that the latter markers were markedly reduced at E9.5, this reflects the absence of the developing labyrinthine layer observed in these mutants. However, the early change seen in *Syna* expression at E7.5 occurs prior to the morphological establishment of SynT and the labyrinth, suggesting that it is a direct effect of methylation deficiency. In contrast, while *Ascl2* mKO trophoblast also displays a reduction in *Syna* expression at E9.5 due to a reduction of the labyrinthine layer (Oh-McGinnis et al., 2011), no difference is detected at E7.5 (Figure S6D).

As SCML2 was not sufficient to drive cell adhesion defects, we asked whether it was necessary for the phenotype, in combination with other methylation-dependent expression changes. We therefore used CRISPR/Cas9 gene editing to delete *Scml2* in TKO TSCs (Figure S6E). Strikingly, both *Scml2* KO clones that were established displayed a distinct morphology from the parental TKO TSCs, closely resembling the WT colony arrangement (Figure 6E). TKO *Scml2* KO clones also showed a restored ability to attach to cell culture wells comparable with WT levels (Figure 6F). This was not due to changes in cell differentiation state, as cells maintained expression of TSC markers and did not show upregulation of differentiation markers (Figure 6G and data not shown). Loss of SCML2 did not rescue the rise in *Cdh2* expression seen in TKO TSCs (Figure 6G). We then asked whether loss of SCML2 was rescuing the cell adhesion defect via modulation of some of the other DE genes identified in mDKO trophoblast. None of the nine genes tested (e.g., *Spry1* and *Itga7*) showed significant differences between TKO TSCs and the TKO *Scml2* KO clones (Figure 6G), suggesting that *Scml2* acts through an independent pathway. Impairment of cell adhesion in TKO TSCs may therefore require both SCML2-dependent pathways together with other methylation-dependent alterations seen in vivo. This is in line with SCML2 overexpression being insufficient to drive cell adhesion changes (Figure S6B).

## Discussion

Our phenotypic, molecular, and functional analyses show that, contrary to previous reports (Sakaue et al., 2010), DNA methylation is essential for early trophoblast development. In particular, our work demonstrates that maternal methylation (and its maintenance during pre-implantation development) plays a major regulatory role in trophoblast differentiation and function. This encompasses the need for controlling imprinted genes (*Ascl2*), but also non-imprinted genes, as demonstrated by the specific example of *Scml2*, which we have shown to affect *Syna* expression and cell adhesion. Moreover, methylation at the *Scml2* promoter is specifically maintained in the EPC and lost in the epiblast (Figure S6A), highlighting the need for targeting DNA methylation to the trophoblast compartment at specific loci. Loss of imprinting at methylation-dependent loci other than *Ascl2* is also known to have an impact on placental function at later developmental stages (Tunster et al., 2013), further emphasizing the importance of maternal methylation.

Although non-imprinted oocyte methylation marks have been generally associated with brain- and testis-linked genes (Rutledge et al., 2014), we have identified critical trophoblast genes that are regulated through maternal DNA methylation (directly or indirectly). Deregulated genes were enriched for signal transduction and regulators of Ras and Rho family GTPases, which are implicated in cell adhesion and migration. Along with the decreased *Cdh1* expression seen in E9.5 DHet trophoblast (Figure 3C), these alterations are in line with the cellular adhesion phenotype seen in mDKO TGCs. Interestingly, links between DNA methylation and EMT have been described in human trophoblast cell lines (Chen et al., 2013a, Chen et al., 2013b). Epigenetic regulation of mouse trophoblast migration and invasion has also been described through the action of the histone lysine demethylase LSD1 (Zhu et al., 2014). Similar to some of our observations, LSD1 depletion in TSCs leads to early differentiation onset, which disrupts their epithelial morphology and increases cell migration and invasion. However, we only found one gene (*Reep6*) in common between mDKO DE genes and LSD1-regulated genes, suggesting that separate epigenetic mechanisms act on different pathways to regulate the crucial processes of cell adhesion and control of invasive behavior in trophoblast.

We uncovered *Scml2* as a putative methylation-controlled gene that is kept silent in the trophoblast lineage to allow for appropriate control of cell adhesion and *Syna* expression. The relatively low methylation levels of *Scml2* in Ctrl EPCs suggest that other mechanisms aid in its silencing. We also cannot completely exclude the possibility that *Scml2* expression is indirectly controlled by DNA methylation. In the male germline, SCML2 regulates PRC1-dependent ubiquitination of histone H2A either positively or negatively, in a context- and target-dependent manner (Hasegawa et al., 2015). SCML2 may therefore regulate the expression of genes involved in the control of cell adhesion and migration, as well as of *Syna*. Notably, based on our *Scml2* overexpression experiment, hypomethylation of *Scml2* is not sufficient to drive the adhesion defects, implicating maternal DNA methylation more widely in the regulation of cell adhesion and migration, as discussed above. Interestingly, human SCML2 interacts with SFMBT2 (Zhang et al., 2013), another PcG protein whose ortholog in mouse is essential for placental development (Miri et al., 2013). *Sfmbt2* is a paternally expressed imprinted gene, but silencing of the maternal allele is independent of DNA methylation (Okae et al., 2012). However, the epigenetic control of *SCML2* during human pre-implantation development appears to differ from that of the mouse, as the syntenic region to the DMR that we identified (also at a TSS and CGI in the human) is largely unmethylated in human oocytes and morulae (Figure S6F) (Guo et al., 2014). Differences in placental morphology and organization may ultimately be driven by such gene-regulatory changes throughout evolution. Notably, the X chromosome is particularly enriched in trophoblast-associated genes and has been argued to be an important driver of placental evolution and speciation (Hemberger, 2002). Along with this argument, it is interesting to note that *Scml2* is X-linked exclusively in eutherians.

Our study highlights a critical and widespread role of oocyte methylation in the development of the placenta. Although we cannot completely rule out that other imprinted genes are involved in the early mDKO phenotype we describe, their known placental roles (Tunster et al., 2013) and our data strongly suggest that additional, non-imprinted methylation marks also control trophoblast development. Interestingly, when we measured human oocyte and morula methylation at regions syntenic to our mouse DMRs, we found that CpGs within these regions are generally hypermethylated when compared with the rest of the genome (Figure S6G). It is tempting to speculate that conservation of methylation at these sites is relevant for human placental development, and that oocyte methylation evolved to play a major role in the trophoblast lineage.

## Experimental Procedures

### Mouse Lines and Tissue Preparation

All experimental procedures were performed under licenses by the Home Office (UK) in accordance with the Animals (Scientific Procedures) Act 1986. Mice carrying conditional deletions of both *Dnmt3a* and *Dnmt3b* (Okano et al., 1999), or of *Dnmt3a* alone, and with or without a *Zp3-Cre* transgene were crossed by natural mating. Female mice heterozygous for an *Ascl2-lacZ* allele (Tanaka et al., 1999) were crossed to WT males. For immunofluorescence, E7.5 implantation sites were fixed with 4% paraformaldehyde (PFA) and processed as for routine paraffin histology. For RNA and DNA isolation, trophoblast and epiblast tissues were dissected from E7.5 or E9.5 conceptuses and snap-frozen.

### Tissue Culture and *Scml2* Overexpression/Knockout

Blastocyst-derived TS-EGFP cells (a kind gift from Dr. Janet Rossant) or WT/TKO TSCs derived from nuclear transfer embryos (Sakaue et al., 2010) were cultured under routine conditions (20% fetal bovine serum, 1 mM Na-pyruvate, penicillin/streptomycin, 50 μM 2-mercaproethanol, 25 ng/ml basic FGF (Sigma) and 1 μg/ml heparin in RPMI 1640, with 70% of the medium pre-conditioned on embryonic feeder cells). For *Scml2* overexpression, the open reading frame of the isoform that starts at the intragenic CGI-associated TSS was cloned via Gateway cloning (Life Technologies) into a PiggyBac vector and sequence-verified. This construct or the empty vector were co-transfected with a PiggyBac transposase plasmid using Lipofectamine 2000 (Life Technologies) or Fugene 6 (Promega), according to the manufacturer's instructions. Integrants were selected with 5 μg/ml blasticidin S, after which cells were switched to TS cell differentiation medium (unconditioned medium without basic FGF or heparin) for the indicated times. For CRISPR/Cas9-mediated deletion of *Scml2*, TKO TSCs were co-transfected with pCAG-cGFP-blasticidin and pCas9.2A.GFP/Puro (Ran et al., 2013) harboring guide RNAs flanking exon 3 (Figure S6D): GTTCATCCCTAGGCAATTAT, CAGGGATGTTTGCAACGTGC. After 48 hr of blasticidin selection, single cells were sorted by flow cytometry and left to grow for 10–14 days before genotyping (see Supplemental Experimental Procedures, Primers).

### Histology and Immunofluorescence

Paraffin sections were deparaffinized with Histo-Clear and dehydrated through an ethanol series, followed either by standard H&E staining or antigen retrieval by boiling slides for 30 min (in 1 mM EDTA, 0.05% Tween 20, pH 8) and cooling at room temperature for 20 min. After blocking with 1% BSA overnight, sections were incubated with a rabbit monoclonal anti-CDX2 antibody (EPR2764Y, Novus Biologicals, 1:250 dilution) for 2 hr. Secondary detection was done with an AlexaFluor 488 anti-rabbit antibody (Life Technologies, 1:500 dilution). TSCs were fixed with 4% PFA, permeabilized with Triton X-100, blocked as above, and labeled with a mouse anti-CDH1 antibody (BD 610181, BD Biosciences, 1:400 dilution).

### Cell Adhesion Assay

Adhesion of TSCs to cell-culture wells was performed using the Vybrant cell adhesion assay kit (Life Technologies) according to the manufacturer's instructions, with the following details/modifications: 10^5^ cells were plated per well of a 96-well tissue culture plate, either uncoated or coated with laminin, and left to attach for 2 hr in serum-free RPMI medium.

### RNA/DNA Isolation and Bisulfite Conversion

Genomic DNA and RNA were isolated from the same samples using the Allprep DNA/RNA Kit (Qiagen). RNA was DNase-treated with the DNA-free kit (Life Technologies). DNA was bisulfite-converted using the Imprint DNA Modification Kit (Sigma).

### RT-qPCR

For RT-qPCR analysis, total RNA was reverse transcribed by random priming using the RevertAid First Strand cDNA Synthesis Kit (Thermo Scientific). qPCR was performed using Brilliant III Ultra-Fast SYBR Green qPCR Master Mix (Agilent Technologies) or Mesa Blue qPCR MasterMix Plus (Eurogentec) using gene-specific primers (see Supplemental Experimental Procedures).

### Sequenom MassARRAY

PCR was performed on bisulfite-converted DNA using HotStarTaq DNA Polymerase (Qiagen) and target-specific primers (see Supplemental Experimental Procedures); samples were processed using the “T” Cleavage MassCLEAVE Reagent Kit (Agena Bioscience) and subjected to MALDI-TOF analysis using the MassARRAY system, according to the manufacturer's instructions.

### RNA-Seq Library Generation and Sequencing

mRNA was purified from 45–400 ng of total RNA using a Dynabeads mRNA Purification Kit (Life Technologies). For each genotype, three strand-specific libraries were generated from single EPCs using the ScriptSeq v2 RNA-Seq Library Preparation Kit (Epicentre), according to the manufacturer's instructions. Indexed libraries were pooled and sequenced on an Illumina HiSeq 2000 or 2500 platform using 50 bp (*Dnmt3* libraries) or 100 bp (*Ascl2* libraries) single-end reads. Reads were trimmed using “Trim Galore!” and mapped to the NCBIM37 genome assembly using TopHat (Trapnell et al., 2009).

### BS-Seq Library Generation and Sequencing

Whole-genome BS-seq libraries were prepared from 25–70 ng of genomic DNA, using a post-bisulfite adaptor tagging protocol, as previously described (Peat et al., 2014). In brief, first-strand synthesis was performed on bisulfite-converted DNA using biotin-tagged random primers containing part of the Illumina-compatible 5′ adaptor sequence. After biotin capture using Dynabeads (Life Technologies), second-strand synthesis was performed using random primers containing part of the Illumina-compatible 3′ adaptor sequence. DNA was then eluted from the beads and libraries amplified using Phusion (New England Biolabs). Libraries were sequenced on an Illumina HiSeq 2500 platform using 100-bp paired-end reads. Reads were trimmed using “Trim Galore!” and mapped to the NCBIM37 genome assembly using bismark (Krueger and Andrews, 2011).

### Bioinformatics Analyses

All data analyses were performed using Seqmonk and/or R scripts. Differential gene expression analysis was performed with DEseq (Anders and Huber, 2010), using a 1% false discovery rate cut-off and minimum fold change of 2. GO analysis was performed using topGO. To measure DNA methylation at given genomic features, methylation calls from both strands at CpG sites were pooled; regions that had at least three CpGs covered by at least five reads were selected and the average CpG methylation value per region calculated. Promoters were defined as −1 kb to +500 bp relative to each TSS; CGI annotation was from Illingworth et al. (2008). For DMR detection, methylation calls from running windows containing five CpGs within 1 kb were pooled; significant differences were determined using a Fisher test and a Benjamini-Hochberg corrected p value cut-off of 0.01. Significantly different windows within 200 bp were merged into DMRs, and only DMRs with a methylation difference larger than 20% were kept. LiftOver was used to identify regions of synteny to the identified DMRs in human. Of 4,084 syntenic regions identified, 353 were covered by at least five reads in human oocyte and morula reduced representation bisulfite sequencing data (Guo et al., 2014) and used for further analysis.

## Author Contributions

M.R.B., W.D., M.H., and W.R. designed the study and experiments. M.R.B. performed histology/immunofluorescence, RT-qPCR, TSC experiments, and bioinformatics analyses. M.K. performed RNA-seq, BS-seq, RT-qPCR, and Sequenom experiments. V.P. performed CRISPR/Cas9 experiments. A.B.B. and L.L. isolated tissue from *Ascl2* KOs. M.C. and E.F. prepared histological sections. W.D. isolated tissue from *Dnmt3* KOs. M.H. performed TSC experiments. S.J.C. interpreted RNA-seq and RT-qPCR data. M.R.B. wrote the manuscript with all other authors.

## Accession Numbers

The accession number for the RNA-seq and BS-seq data reported in this paper is GEO: GSE66049.

## Supplemental Information

Document S1. Supplemental Experimental Procedures, Figures S1–S6, and Tables S2 and S3

Table S1. Differentially Expressed Genes in All mDKO Genotypes versus Ctrl EPCs, Related to Figure 1

Document S2. Article plus Supplemental Information
