# Supplementary material for: Maternal DNA Methylation Regulates Early Trophoblast Development
Source: Dev Cell. 2016 Jan 25;36(2):152–63. doi: 10.1016/j.devcel.2015.12.027 (PMC4729543; doi:10.1016/j.devcel.2015.12.027)
Supplement: Document S1. Supplemental Experimental Procedures, Figures S1–S6, and Tables S2 and S3 [file mmc1.pdf]

Developmental Cell, Volume *36*

Supplemental Information

Maternal DNA Methylation Regulates Early Trophoblast Development

Miguel R. Branco, Michelle King, Vicente Perez-Garcia, Aaron B. Bogutz, Matthew Caley, Elena Fineberg, Louis Lefebvre, Simon J. Cook, Wendy Dean, Myriam Hemberger, and Wolf Reik

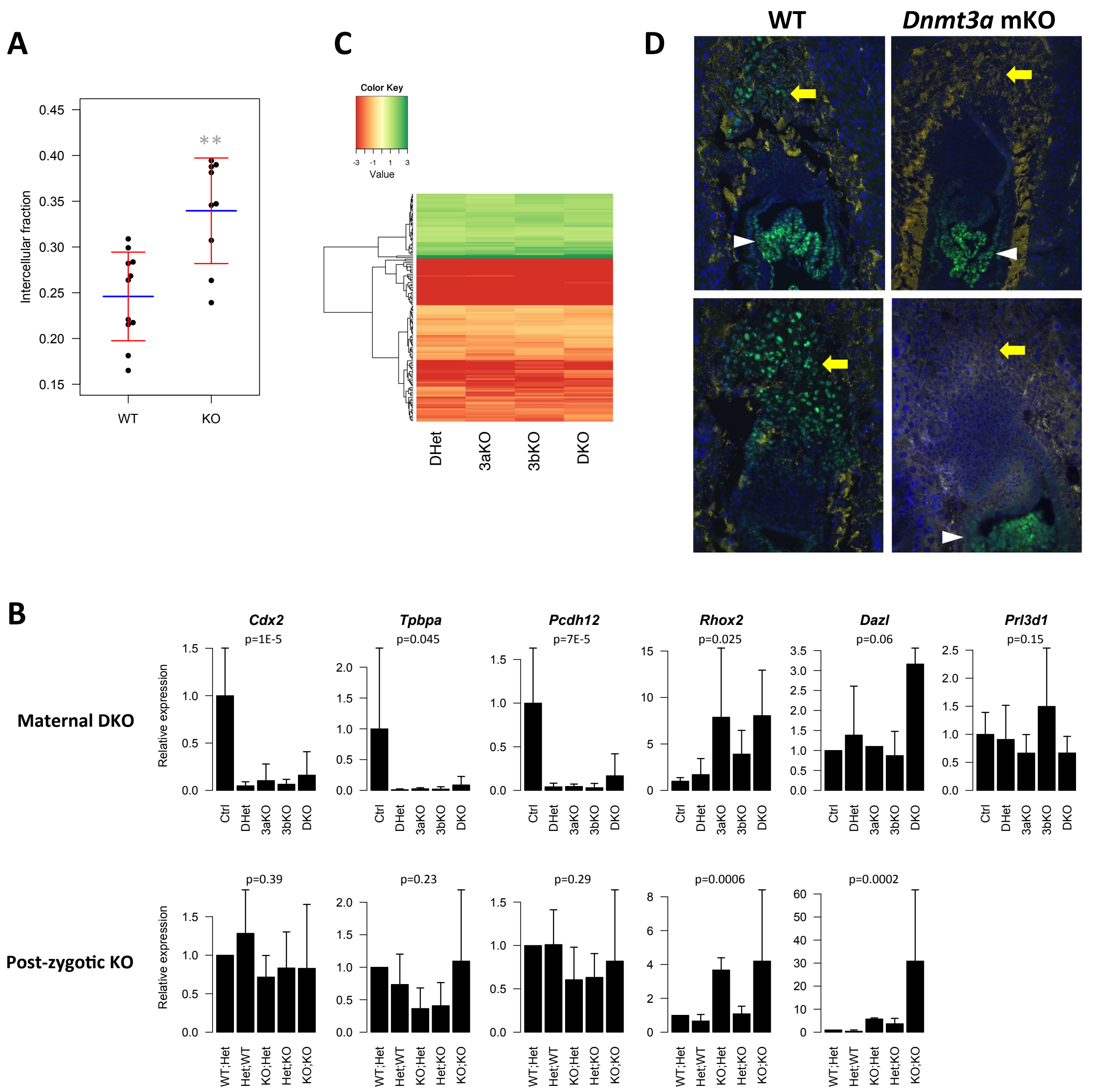

Figure S1 (related to Figure 1) – Gene expression alterations in Dnmt3 KO EPCs. A) The amount of extracellular space at the TGC layer was measured from histological sections (Fig. 1C) and normalised to the total area, showing that *Dnmt3a* mKO trophoblast sections show significantly increased in spacing between cells (**p<0.01, t-test). B) RT-qPCR analysis in oocyte and post-zygotic *Dnmt3* deletion E7.5 EPCs. Post-zygotic deletion was generated by crossing mice heterozygous for both *Dnmt3* genes; genotypes under each bar refer to *Dnmt3a* and *Dnmt3b*, respectively (e.g., Het;KO signifies *Dnmt3a*$^{+/-}$;*Dnmt3b*$^{-/-}$). P-values refer to the effect of genotype as analysed by ANOVA. C) Expression differences to Ctrl EPCs across all mDKO DE genes. Log2 fold changes in each mDKO genotype relative to Ctrl EPCs are displayed as a heatmap. D) Immunofluorescence for CDX2 (green) in two sections from E7.5 wildtype or *Dnmt3a* mKO conceptuses. Sections were counterstained with DAPI (blue). Yellow staining is from autofluorescence of blood cells. In the wildtype, expression of CDX2 is visible both in the extraembryonic ectoderm (white arrowhead), where the TSC niche lies, and in the EPC (yellow arrow). In *Dnmt3a* mKO expression of CDX2 is specifically lost in the EPC, remaining high in the extraembryonic ectoderm. Error bars represent standard deviations.

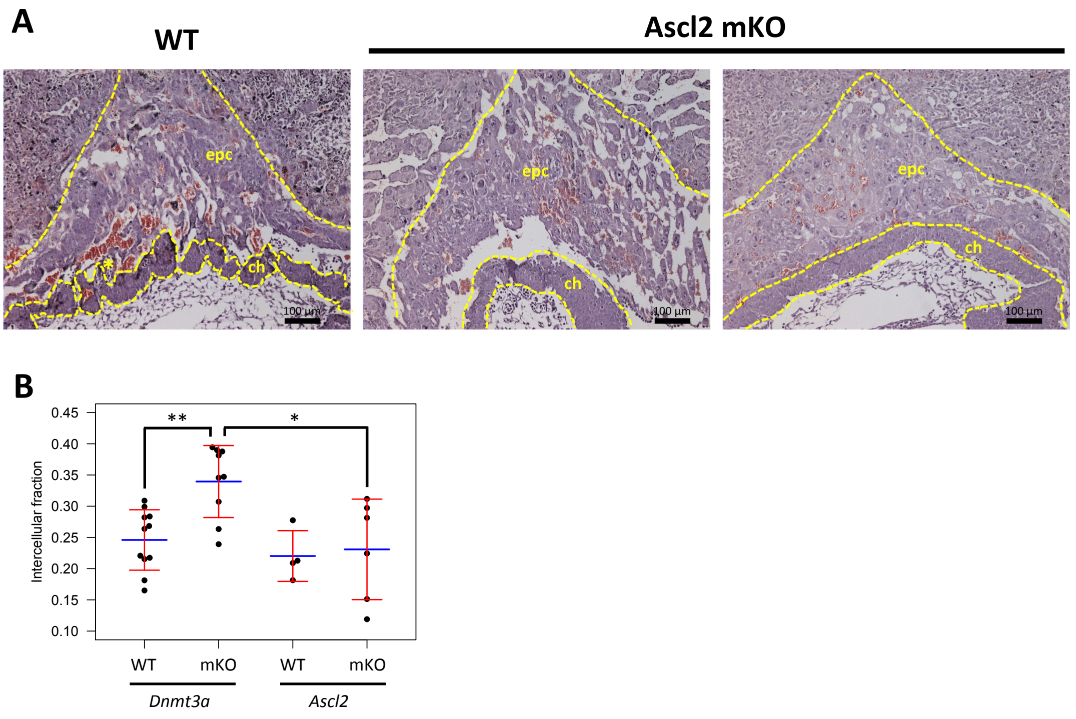

Figure S2 (related to Figure 2) – Histological analysis of *Ascl2* mKO E9.5 trophoblast. A) Hematoxylin and eosin staining of paraffin sections shows that *Ascl2* mKO trophoblast lacks the labyrinthine layer that is otherwise seen developing in WT trophoblast (marked by an asterisk); the TGC layer is expanded but, unlike *Dnmt3a* mKOs, does not have a pronounced reduction in tissue density when compared to WT TGCs; ch – chorion, epc – ectoplacental cone. B) The amount of extracellular space at the TGC layer was measured and normalised to the total area, showing that *Ascl2* mKO sections do not have increased spacing between cells. *p<0.05, **p<0.01 – ANOVA followed by post-hoc tests comparing *Dnmt3a* mKO with other genotypes. Error bars represent standard deviations.

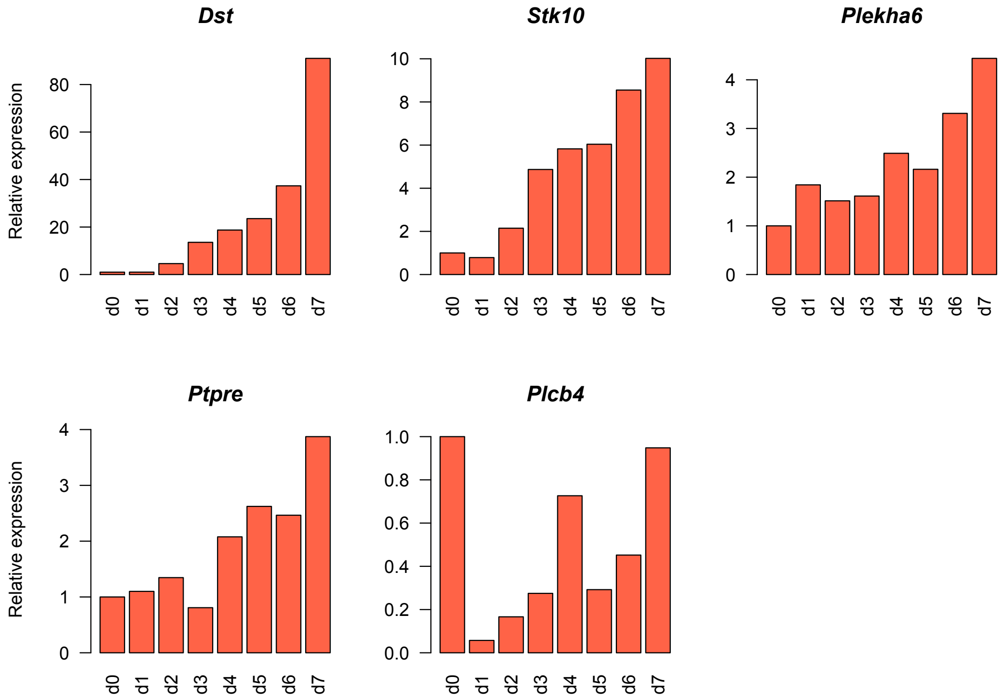

Figure S3 (related to Figure 3) – RT-qPCR analysis of *Dnmt3* mDKO DE genes during TSC differentiation. TSCs were differentiated *in vitro* for 7 days. Genes with increased expression during *in vivo* differentiation (Fig. 3A) also show a differentiation-dependent profile *in vitro*.

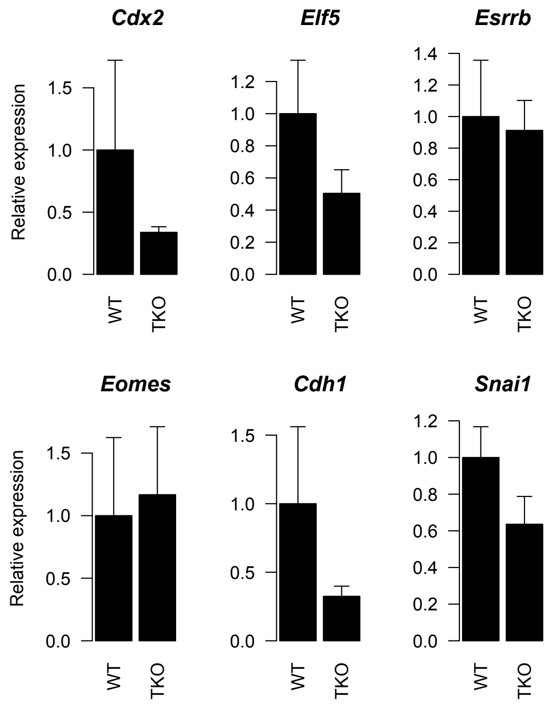

Figure S4 (related to Figure 4) – Additional RT-qPCR analysis of TKO TSCs. TKO TSCs show no significant differences in the expression of key TSC markers (*Cdx2*, *Elf5*, *Esrrb*, *Eomes*) or the EMT markers *Cdh1* and *Snai1*. Error bars represent standard deviations.

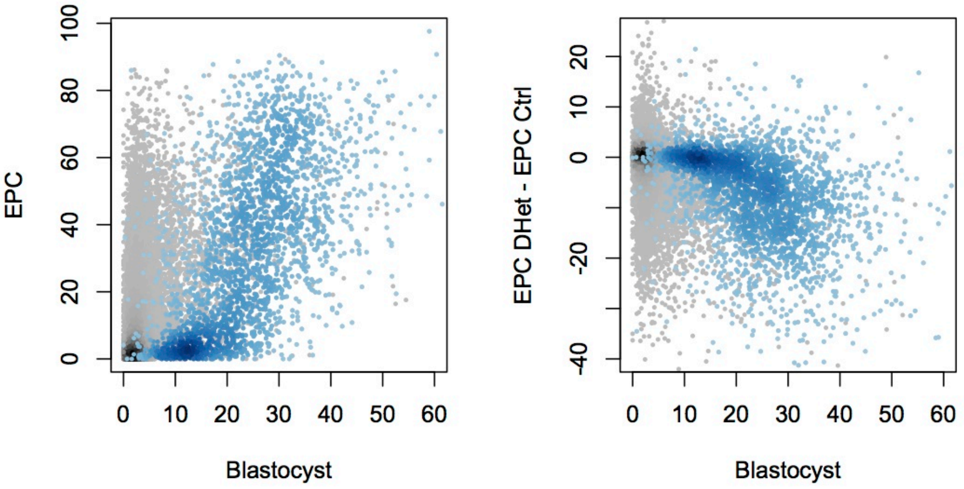

Figure S5 (related to Figure 5) – Hypomethylation in DHet embryos is not preferentially derived from reprogramming-resistant loci. Blastocyst methylation data at CGIs (Kobayashi et al. 2012) was plotted against methylation levels in Ctrl EPCs (left) or the difference between DHet and Ctrl EPCs (right). CGIs with more than 50% methylation in oocytes are highlighted in blue and show that nearly all CGIs methylated in blastocyst are carried over from the oocyte. However, EPCs display de novo methylated CGIs as well. Hypomethylation in DHet EPCs occurs in both reprogramming-resistant and de novo methylated CGIs.

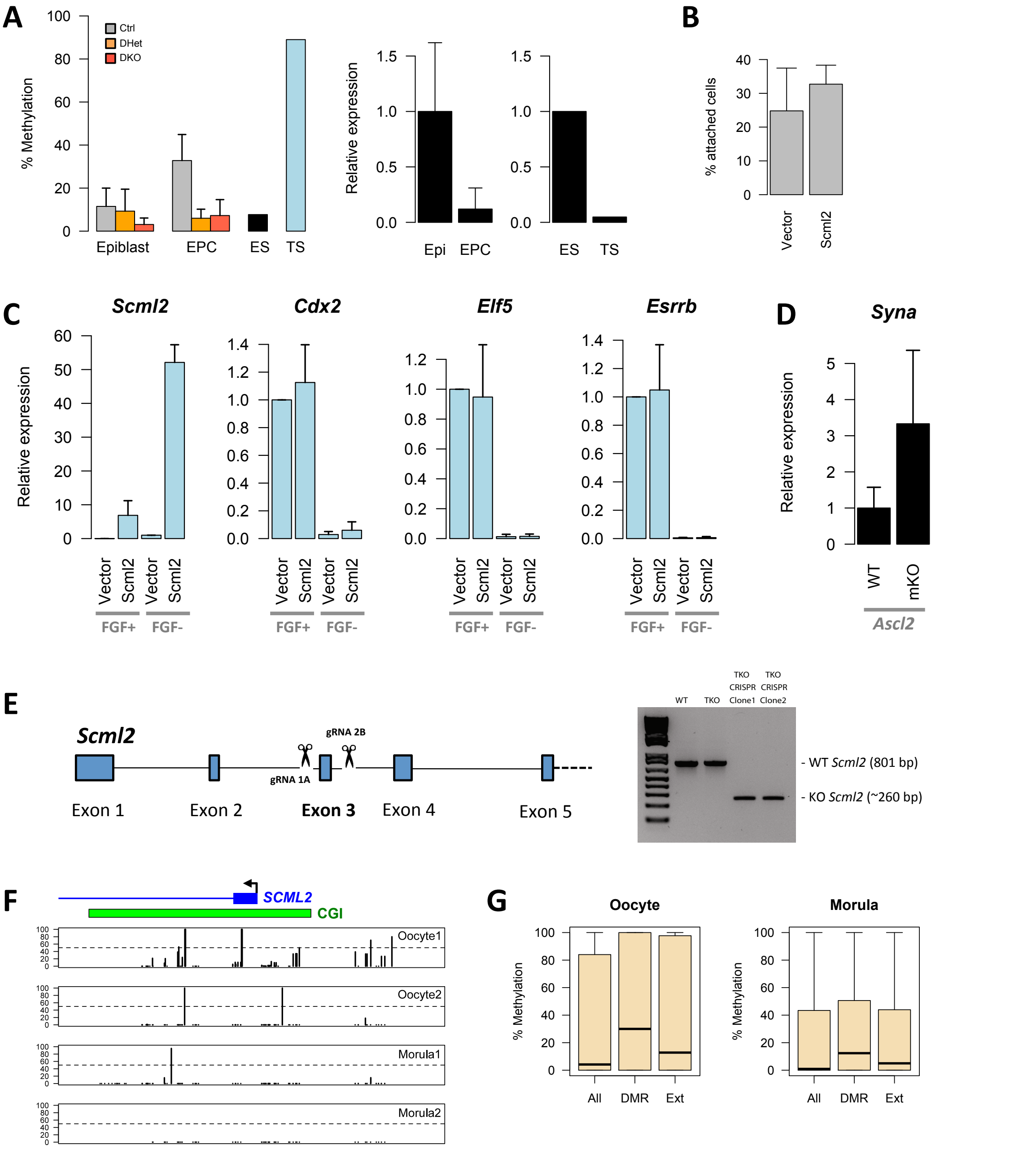

Figure S6 (related to Figure 6) – *Scml2* regulation and role in early development. A) *Scml2* methylation (left; amplicon CpGs 8-10) and expression (right) show asymmetry between E7.5 EPC and epiblast, and between TSCs and ESCs. B) Measurement of TSC adhesion to uncoated tissue culture wells shows that *Scml2* overexpression is insufficient to drive adhesion defects. C) Additional RT-qPCRs in *Scml2*-overexpressing TSCs, grown under stem cell (FGF+) or differentiation (FGF-) conditions. D) *Syna* expression in WT and *Ascl2* mKO E7.5 EPCs. E) CRISPR/Cas9 strategy for knocking out *Scml2* in TKO TSCs and corresponding genotyping PCRs. F) DNA methylation in human oocytes and morulae (Guo et al. 2014) at the syntenic region to the *Scml2*-associated DMR; no methylation is seen in morulae. G) Comparison of CpG methylation between the whole genome ('All'), at regions of synteny with all mDKO DMRs ('DMR') and at DMR regions extended up to 400bp to include adjacent genomic regions ('Ext'). Overall, mDKO DMRs are hypermethylated in human oocytes and morulae; extending the DMRs dampens the difference, demonstrating local specificity of the hypermethylation. Bar plot error bars represent standard deviations.

SUPPLEMENTARY TABLES

Table S1 (related to Figure 1) – Differentialy expressed genes in all mDKO genotypes versus Ctrl EPCs. Log2 fold-change and p values refer to the comparison of DHet with Ctrl EPCs. P values are corrected for multiple comparisons. Genes that are also differentially expressed in Ascl2 mKO EPCs are indicated.

Table S2 (related to Figure 2) – Gene ontology terms associated with Dnmt3-specific DE genes. The top 20 significant GO terms are shown. Benjamini-Hochberg correction of p values was applied.

GO ID	GO Term	n Genes	Enrichment	p value
GO:0043087	regulation of GTPase activity	13	9.49	6.80E-06
GO:0033124	regulation of GTP catabolic process	13	9.42	6.80E-06
GO:0032318	regulation of Ras GTPase activity	11	11.58	7.20E-06
GO:0030811	regulation of nucleotide catabolic process	13	8.61	7.20E-06
GO:0033121	regulation of purine nucleotide catabolic process	13	8.61	7.20E-06
GO:0009118	regulation of nucleoside metabolic process	13	8.55	7.20E-06
GO:0046578	regulation of Ras protein signal transduction	12	8.89	1.20E-05
GO:0007265	Ras protein signal transduction	13	8.02	1.20E-05
GO:1900542	regulation of purine nucleotide metabolic process	14	7.14	1.20E-05
GO:0006140	regulation of nucleotide metabolic process	14	7.07	1.20E-05
GO:0051056	regulation of small GTPase mediated signal transduction	12	8.45	1.90E-05
GO:0006184	GTP catabolic process	13	6.63	7.00E-05
GO:1901069	guanosine-containing compound catabolic process	13	6.57	7.00E-05
GO:0046039	GTP metabolic process	13	6.50	7.00E-05
GO:0023051	regulation of signaling	27	3.01	7.00E-05
GO:0010646	regulation of cell communication	27	3.00	7.00E-05
GO:1901068	guanosine-containing compound metabolic process	13	6.37	8.20E-05
GO:0009966	regulation of signal transduction	25	3.14	8.60E-05
GO:0031329	regulation of cellular catabolic process	14	5.60	0.00011
GO:0009653	anatomical structure morphogenesis	25	3.09	0.00011

Table S3 (related to Figure 2) – Gene ontology terms associated with Ascl2-specific DE genes. The top 20 significant GO terms are shown. Benjamini-Hochberg correction of p values was applied.

GO ID	GO Term	n Genes	Enrichment	p value
GO:0048583	regulation of response to stimulus	65	2.21	3.70E-06
GO:0044699	single-organism process	185	1.30	2.20E-05
GO:0051239	regulation of multicellular organismal process	51	2.19	0.00012
GO:0048731	system development	72	1.86	0.00012
GO:0051179	localization	87	1.72	0.00012
GO:0044763	single-organism cellular process	168	1.32	0.00012
GO:0044765	single-organism transport	62	1.96	0.00018
GO:0009966	regulation of signal transduction	50	2.15	0.00024
GO:0023051	regulation of signaling	53	2.02	0.00052
GO:0010646	regulation of cell communication	53	2.01	0.00052
GO:0007275	multicellular organismal development	77	1.72	0.00052
GO:0009611	response to wounding	25	3.09	0.00062
GO:0048519	negative regulation of biological process	68	1.78	0.00062
GO:0048518	positive regulation of biological process	74	1.72	0.00062
GO:0006810	transport	70	1.74	0.00088
GO:2000026	regulation of multicellular organismal development	36	2.35	0.00093
GO:0050793	regulation of developmental process	42	2.18	0.00093
GO:0044767	single-organism developmental process	82	1.63	0.00093
GO:0050896	response to stimulus	121	1.42	0.00093
GO:0032502	developmental process	82	1.62	0.00095

SUPPLEMENTARY EXPERIMENTAL MATERIALS

Primers

Target	Primer pair sequences
RT-qPCR	
Ascl2	AGCCCGATGGAGCAGGAG<br>CCGAGCAGAGGTCAGTCAGC
Atp5b	GGCCAAGATGTCCTGCTGTT<br>GCTGGTAGCCTACAGCAGAAGG
Cdh1	TGACTCGAAATGATGTGGCT<br>GCTGCCTTCAGGTTTTCATC
Cdh2	CTTCAGGCGTCTGTGGA<br>CTGAATTTCACATTGAGAAGGG
Cdx2	AGTGAGCTGGCTGCCACACT<br>GCTGCTGCTGCTTCTTCTTGA
Cebpa	TGGACAAGAACAGCAACGAG<br>TCACTGGTCAACTCCAGCAC
Dazl	CTTACATGCAGCCTCCAACC<br>GCGGTGGCATCTGGTAGTTA
Dst	ATTCAAGAGTTCATGGACCTACG<br>CCCGTGCTCAGAATTCTCTTTA
Elf5	ATTCGCTCGCAAGGTTACTCC<br>GGATGCCACAGTTCTCTTCAGG
Eomes	CCTGGTGGTGTTTTGTTGTG<br>TTTAATAGCACCGGGCACTC
Esrrb	AGTACAAGCGACGGCTGGAT<br>CCTAGTAGATTCGAGACGATCTTAGTCA
Itga7	CTGCTGTGGAAGCTGGGATTC<br>CTCCTCCTTGAACTGCTGTCG
Hspcb	GCTGGCTGAGGACAAGGAGA<br>CGTCGGTTAGTGGAATCTTCATG
Pcdh12	GAAGAGCTGTCGAGCCTGTT<br>GTGAGGGGCAATGACAATCT
Pl1	GACATTAAGGGCAGAAACCTTG<br>GTCCAGACCAAGCAGGGTAG
Plcb4	TAGAGGATGAGCAAGCATGG<br>TGAATATTGCGCTCTTCAGC
Plekha6	GTGAAAGGAGTTAGAGGCAGCA<br>GTGGACAGAAGGGCTCCAT
Ptpre	CCCACGACCCTCCCT<br>CAAGGGGAAATGAGGGCTA
Rhox2	AGAGCTTCAATGTGCTGCAA<br>CAAAAACCATTCCTGCACTG
Scml2	ATCTTCCCAGTTGGATGGTG<br>CTGGGGCCTCTTCTTCATTT
Snai1	GAGTTGACTACCGACCTTGC<br>AAGGTGAACTCCACACACG
Snai2	CATTAGAACTCACACTGGGGAA<br>TTTACATCAGAGTGGGTCTGC
Spry1	GGTCATAGGTCAGATCGGGTC<br>CTTGCCACACTGTTCGCAG
Stk10	ACACCCTCCAAGTGGTCTGT<br>GAGCCTTATTGCTGGTGACTCT
SynA	CCTCACCTCCCAGGCCCCTC<br>GGCAGGGAGTTTGCCCACGA
SynB	TCCGGAAAGGGACCTGCCCA<br>CAGCAGTAGTGCGGGGTGCC
Tpbpa	CGGAAGGCTCCAACATAGAA<br>GGCTGTGGTTTGTTTTCCTC
Twist1	AGACCTAGATGTCATTGTTTCCAGA<br>TTGTGAATTTGGTCTCTGCTCTT

Twist2	TCTCAGCTACGCCTTCTCC<br>TGAGATGTGCAGGTGGGT
Zeb1	GAGGTGACTCGAGCATTTAGAC<br>TCTGAATTTGCTTCTACCACAGT
Zeb2	GGAGGAAAAACGTGGTGAAC<br>GGGTTTGCAAGGCTATCATC
Sequenom	
Scml2 TSS	AGGAAGAGAGGTATTTGGGGTAAAGTTTTTAGGGG<br>CAGTAATACGACTCACTATAGGGAGAAGGCTTAAATCCTAAACTCAAACTCCACCC
Genotyping	
Scml2	AGCACTTCTCCCTCCCTTTT<br>CAAGGCTCAAGGCAAAAATC
